# Exon sequencing of the alpha-2-globin gene for the differential diagnosis of central cyanosis in newborns: a case report

**DOI:** 10.1186/s12887-019-1601-9

**Published:** 2019-07-03

**Authors:** Chungwoo Shin, Mee Hong, Myungshin Kim, Jung Hyun Lee

**Affiliations:** 1 Department of Pediatrics, St. Vincent’s Hospital, College of Medicine, The Catholic University of Korea, 93-6 Ji-dong, Paldal-gu Suwon-si, Gyeonggi-do 16247 South Korea; 2 Department of Laboratory Medicine, College of Medicine, The Catholic University of Korea, Seoul, South Korea; 3 Catholic Genetic Laboratory Center, College of Medicine, The Catholic University of Korea, Seoul, South Korea

**Keywords:** Hemoglobin M, Cyanosis, Newborn, Methemoglobin

## Abstract

**Background:**

Cyanosis is usually associated with serious conditions requiring urgent treatment in the neonatal intensive care unit (NICU). Hemoglobin M (Hb M) disease is one type of congenital methemoglobinemia characterized by cyanosis. Among these variants, α-globin chain mutations such as Hb M Boston present cyanosis from birth while other variants usually manifest later in life.

**Case presentation:**

We report a case of a male newborn with cyanosis apparent since birth. Surprisingly, his respiratory and hemodynamic status including normal arterial blood oxygen saturation was stable, but oxygen saturation on pulse oximetry did not increase after 100% supplemental oxygen was started. In addition to routine pulmonary and cardiologic evaluation, further evaluation for dyshemoglobin was conducted; α2-globin gene sequencing showed a single-point variant causing Hb M Boston. Methemoglobin (MetHb) level estimated by co-oximetry was normal. After a 14-day stay in the NICU, the patient remained respiratory and hemodynamically stable without supplemental oxygen except for cyanosis.

**Conclusions:**

Hb M disease is a benign disease and does not require any treatment whereas acquired methemoglobinemia is a potentially fatal condition. Neonatologists should be aware that low oxygenation status on pulse oximetry in the face of normal arterial blood saturation values might indicate the possibility of Hb M disease in early neonatal cyanosis, irrespective of MetHb value.

## Background

Hemoglobin M (Hb M) is one of the causes of inherited methemoglobinemia. Methemoglobin (MetHb) refers to the oxidized form of hemoglobin. Hb M caused by a mutation in α-, β-, or γ-globin can lead to spontaneous oxidation of the ferrous ion in the heme and cannot transport or release oxygen in tissues. Hb M disease causes cyanosis that is unresponsive to oxygen therapy [1, 2]. Some variants might need to be considered in the differential diagnosis of central cyanosis in newborns because they cause cyanosis from birth. Hemoglobin variants by a genetic mutation are different from natural MetHb in the absorption spectrum. Then, co-oximetric measurement may be inaccurate in cases of Hb M [3].

Unlike other types of methemoglobinemia, Hb M disease is a benign disease and does not require any treatment although it reveals a history of lifelong cyanosis. Clinical suspicion and early diagnosis could avoid unnecessary investigations and invasive management for patients and alleviate concern for their family [4, 5]. In this study, we present a male newborn with Hb M Boston and review the literature for Hb M disease since 1961.

## Case presentation

**Fig. 1:**
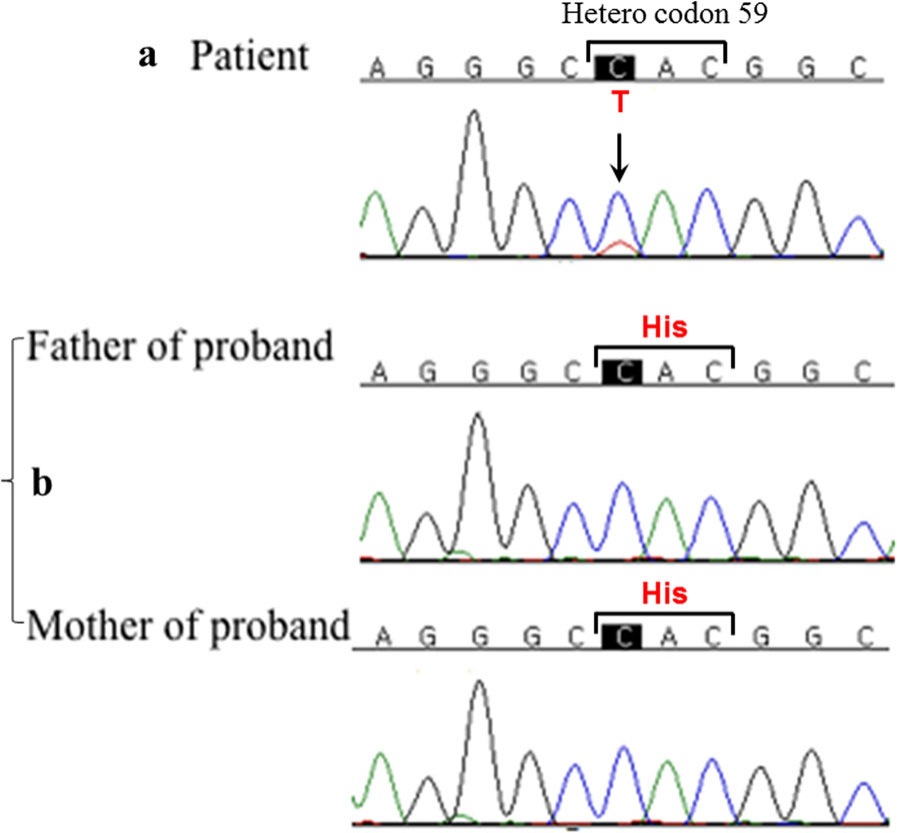
**a** The mutation underlying heterozygous hemoglobin M Boston [*HBA2*:c.175C > T, p.59His > Tyr] was detected through sequencing only in the proband. **b** It was not present in his parents. The analysis demonstrated a de novo mutation occurrence

A male newborn was referred to the neonatal intensive care unit with cyanosis from birth. After applying supplemental oxygen, pre- and postductal saturation was 80%. Tracheal intubation and positive pressure ventilation with high inspired oxygen concentration did not improve the low oxygen saturation (SO_2_) level. The infant weighed 3400 g and was delivered uneventfully at 38 + 6 weeks from a 32-year-old mother. He did not appear to have any signs of respiratory difficulties except for cyanosis. When SO_2_ was 77% according to pulse oximetry, the value of partial pressure of oxygen (PaO_2_) on arterial blood gas analysis was 114 mmHg. His complete blood count and C-reactive protein were normal. After ruling out respiratory and cardiac causes based on chest radiographic and echocardiographic studies, further evaluation for hemoglobin derivatives incapable of binding oxygen was conducted. MetHb level estimated by co-oximetry was found to be normal. In addition, hemoglobin electrophoresis at alkaline pH on agarose gel showed normal age profiles with 82.7% Hb F, 17.2% Hb A1, and 0% Hb S. Exon sequencing was conducted for the α-globin chain of hemoglobin because of the cyanosis at birth. We found a c.175C > T point variant of *HBA2* exon 2, which causes a change of the 59th amino acid from histidine to tyrosine (Fig. 1a). This single-point variant causes Hb M Boston. The genetic evaluation of *HBA2* in his parents was normal for the above mutation (Fig. 1b); this appeared to be a de novo mutation. Gene sequencing of erythroid Krüppel-like factor for thalassemia and CYB5R3 for methemoglobinemia types 1 and 2 was negative.

At discharge, the patient remained respiratory and hemodynamically stable without supplemental oxygen except for cyanosis. At 2 years of age, his weight was 16.5 kg and height was 95.5 cm, placing him in the 99th percentile according to national growth charts for children; however, he remained slightly cyanotic at 83% saturation without supplemental oxygen.

## Discussion

Since chemical characterizations of Hb M were first reported in the late 1950s and early 1960s by Gerald [6, 7], the rare globin chain variants have been investigated by chromatography, electrophoresis, and gene sequencing [1, 2, 5].

This is the first de novo case of Hb M Boston reported from South Korea [8]. Like other types of Hb M disease, Hb M Boston has an autosomal dominant inheritance pattern [5]. Although de novo mutations are not rare events in human genetic diseases, relatively few have been recorded for Hb M variants and α-chain mutants are rarer than β-chain mutants [2, 5, 9–12]. This report clearly describes a de novo mutation that was present in the proband by gene sequencing of *HBA2*. Hb M should be considered in the differential diagnosis of cyanosis in the newborn period, even if no familial cases are detected. Table 1 lists the cases of hemoglobin M disesase with neonatal cynosis presenting immediately after birth.

**Table 1:** Summary of hemoglobin M diseases presenting as neonatal cyanosis

Reference	Form of genetic variation	Hemoglobin subunit	MetHb level estimated by co-oximetry	Hemoglobin variants
Viana et al., 2014 [1]	De novo mutation	Alpha 2	12.5%	Hb M Iwate
Estey et al., 2015 [2]	Familial	Alpha 2	Measuring error	Hb M Boston
Elboraee et al., 2015 [5]	Familial	Alpha 2	Measuring error	Hb M Boston
Upadhye et al., 2015 [11]	Familial	Alpha	13%	Hb M Boston
Alonso-Ojembarrena et al., 2016 [14]	Familial	Gamma	12.3%	Hb M Osaka

*MetHb* methemoglobin

Hemoglobin is constantly being oxidized; however, natural reducing systems such as erythrocyte MetHb reductase maintain the natural MetHb level under 2% [2, 5]. Hb M variants caused by a mutation in α-, β-, or γ-globin make the redox potential of the heme iron more negative; the oxidized heme then becomes more resistant to reduction by erythrocyte MetHb reductases [2, 13]. Consequently, Hb M disease causes methemoglobinemia and contributes to lifelong cyanosis that is unresponsive to oxygen therapy. In particular, the presence of cyanosis in the neonate supports an α-globin chain variant such as Hb M Boston, because of a β-globin mutation present several months later due to low β-chain expression at birth [2].

In this study, the range of SO_2_ on pulse oximetry was 77–83%. Although we applied supplemental oxygen, no significant increase in saturation was seen. By contrast, several estimated saturation values calculated from PaO_2_ using a blood gas analyzer were consistently above 95%. This discrepancy results from the unique effects of MetHb on standard oxygenation assessments and can distinguish it from cyanotic congenital heart disease [2, 4, 5]. MetHb can be measured directly in blood by co-oximetry using multiple wavelengths of light to distinguish not only the fractions of oxyhemoglobin and deoxyhemoglobin, but also MetHb and carboxyhemoglobin [3]. In some cases of Hb M, MetHb level can be underestimated when measured using co-oximetry [2, 5, 11, 14]. Certain mutations in the gene coding for one of the globin chains cause conformational changes in hemoglobin, and their absorbance spectrums can differ from typical MetHb [3]. Therefore, co-oximetry might not be useful to detect the percentage of MetHb arising from congenital variants like Hb M, as demonstrated in our case. In addition, unlike acquired methemoglobinemia with much higher amounts of MetHb, the patients with congenital methemoglobinemia typically have MetHb values of less than 20% that cause clinically obvious cyanosis; however, the patient is otherwise asymptomatic [4, 5].

In this study, the presence of Hb M disease and the pattern of de novo mutation were simultaneously established by DNA sequencing, not by electrophoresis or high-performance liquid chromatography. Because different Hb M variants may have similar profiles using some or all of these techniques, DNA gene sequencing is the definitive method for distinguishing various hemoglobin variants [2, 5].

## Conclusion

Hb M disease is a rare blood disorder, but needs to be considered in newborns with cyanosis or low SO_2_ on pulse oximetry in the face of normal arterial blood saturation regardless of MetHb value. Early diagnosis helps to avoid unnecessary diagnostic approaches and aggressive interventions because the hemoglobin variants show a good prognosis.

## Data Availability

All available data is presented in the main manuscript.

## Abbreviations

Hb M: Hemoglobin M
MetHb: Methemoglobin
PaO_2_: Partial pressure of oxygen
SO_2_: Oxygen saturation
